# Analysis of Mechanical Parameters of Asymmetrical Rolling Dealing with Three Region Percentages in Deformation Zones

**DOI:** 10.3390/ma15031219

**Published:** 2022-02-06

**Authors:** Qilin Zhao, Xianghua Liu, Xiangkun Sun

**Affiliations:** 1 School of Materials Science and Engineering, Northeastern University, Shenyang 110819, China; zhaoql73@163.com; 2 State Key Laboratory of Rolling and Automation, Northeastern University, Shenyang 110819, China; sxk20081647@163.com

**Keywords:** strip asymmetrical rolling, mathematic models, percentage of cross-shear region, friction work, energy consumption, slab method

## Abstract

A series of mathematical models were proposed to calculate the roll force, torque and power for cold strip asymmetrical rolling by means of the slab method, taking the percentages of the forward-slip, backward-slip and cross-shear zones into account. The friction power, plastic work and total energy consumption can be obtained by the models. The effects of variable rolling parameters—such as the speed ratio, entry thickness, friction coefficient and front and back tension—on the process of asymmetrical rolling are analyzed. In all cases, an increase in speed ratio leads to an increase in friction work and its proportions. The increase in entry thickness and deformation resistance causes both friction work and plastic deformation work to increase. The proportion of friction work decreases with increasing deformation resistance, entry thickness, front tension and back tension. In the circumstances of a thin strip being rolled with a large speed ratio, the proportion of friction work could exceed that of plastic deformation work. The concept of a threshold point of friction work was proposed to explain this phenomenon. As an example, threshold points T1, T2, T3 with the effect of the entry thickness and S1, S2, S3 with the effect of the friction coefficient have been obtained by computation. Finally, the experiment of the strip asymmetrical rolling was conducted, and a maximum error of 9.7% and an RMS error of 5.9% were found in the comparison of roll forces between experimental measurement values and calculated ones.

## 1. Introduction

In asymmetrical rolling, the neutral point of the upper roll and lower roll is not aligned vertically due to there being different velocities on the upper and lower surface of the workpiece; this could be caused by the different diameters of the rolls, their different peripheral velocities or the different friction conditions between the workpiece and the rolls. Thus, within the deformation zone and beside the backward-slip zone and the forward-slip zone in a common rolling, there is a unique zone in the asymmetrical rolling process called the cross-shear zone. One obvious feature of the cross-shear zone is that the direction of friction force on the upper and lower surfaces of the strip is reversed.

Since the asymmetrical rolling theory was first introduced [1], extensive experimental investigations and theoretical analyses have been conducted on asymmetrical rolling. The curvature of a workpiece caused by asymmetrical rolling aroused great interest. The curvature of rolled material was experimentally investigated [2], and the effects of different work roll diameters and reduction ratios on the bending of the workpiece were analyzed by the finite element method [3]. Analytical models based on the slab method [4] and the finite element method [5] were built to predict strip curvature.

Since one of the advantages of asymmetrical rolling is that less roll force and less roll torque are required, various models are thus built to investigate the deformation in asymmetrical rolling and to analyze the influences of different rolling parameters. Analytical models were built by the slab method to investigate the deformation mechanism of the sheet at the roll gap during asymmetrical cold strip rolling [6]; an experimental study was also conducted [7], and the stream function method [8] and finite element method [9] were employed in further analysis. The influence of the friction coefficient ratio on shear deformation, rolling pressure and torque was investigated using slab analysis [10]. The plane strain asymmetrical rolling was analyzed by a model based on the slab method that considered the contact arc as the parabola [11]. Analytical models considering the shear stress along the vertical sides of each slab were built to calculate the roll force and torque [12]. An analytical model was built by the slab method to analyze the effects of the work roll radius, roll speed and the friction coefficient on rolling pressure, roll force and roll torque [13], and to study the relationship between the asymmetrical rolling deformation zone configuration and rolling parameters [14]. Analytical models were also built for the numerical study of multi-layer sheet rolling [15,16,17].

Asymmetrical rolling is beneficial to enlarging deformation, resulting in its outstanding capability in thickness reduction. The minimum thickness limit of symmetrical rolling was broken in the asymmetrical rolling experiment [18]. Analytical models were built to investigate the minimum thickness in asymmetrical cold rolling [19,20] by the slab method. A semi-empirical formula was built to calculate the minimum thickness on the basis of experimental and theoretical studies [21]. Analysis of the relationship between the deformation zone configuration and rolling parameters shows that the minimum thickness can be reached when the forward-slip disappears, and the rolling parameters keep the deformation zone configuration as a cross-shear zone and backward-slip zone (C + B), or an all-cross-shear zone (AC) [22]. The softening phenomenon [23] and size effect [24] in asymmetrical rolling of pure copper foil were studied. An analytical model based on the slab method, which considers the percentage of three regions in the plastic deformation zone, was used to study the effect of rolling parameters on the deformation zone configuration, and provided an accurate calculation of roll force and roll torque [25,26]. The main parameters of rolling a particular asymmetric regime were obtained from an asymmetric rolling process simulation for establishing a link between the peripheral speeds of the rolls, pressure and contact length [27].

Asymmetrical rolling technology is beneficial to grain refinement and texture control, so it is used as a method to improve the mechanical properties of materials. Ultrafine grain with an average size of 0.5μm was obtained by asymmetrical cold rolling of an AA1016 aluminum strip [28], and refined surface grain with an average size of ~3μm was obtained by asymmetrical hot rolling of non-magnetic austenitic steel [29]. After a two-time asymmetrical rolling and heat treatment of the AA1050 Al alloy sheet, the R-value of the sheet increased from 0.61 to 1.3 [30]. A single-pass asymmetric rolling was carried out on extra-low-carbon steel to investigate the influence of thickness reduction per pass on texture evolutions [31]. Models (FEM coupled with microstructure evolution models and cellular automata models) were also built to study the microstructure evolution of plates during asymmetrical rolling [32]. The effects of processing parameters of asymmetrical rolling on the mechanical properties of aluminum alloy AA6061 were investigated experimentally [33].

Though various models were built for asymmetrical rolling, most of them were suitable for plate and sheet rolling, and only several models were suitable for thin strip and foil rolling. The deformation behavior of a workpiece in thin strip and foil rolling are different from that in plate and sheet rolling; the friction and the tension play important roles in thin strip and foil rolling. Energy consumption, especially in work used to overcome the friction resistance, is important for the industrial application of asymmetrical rolling technology to thin strip and foil rolling. In this paper, analytical models based on the slab method are proposed for asymmetrical strip rolling, considering the three region (backward-slip zone, forward-slip zone and cross-shear zone) percentages in the deformation zone. The roll pressure, roll force, roll torque and roll power are calculated to obtain the friction work and plastic deformation work. The effects of rolling parameters, such as the speed ratio, entry thickness, front and back tension, the friction coefficient and the deformation resistance of the strip on friction work in asymmetrical thin strip rolling, are analyzed.

## 2. Mathematical Models

### 2.1. Basic Assumptions

To simplify the derivation of analytical models, the following assumptions are employed:

- The rolls are rigid bodies; the strips being rolled are rigid-plastic material.
- The friction coefficients between the strip and the roll are constant, but may be different on the upper and lower surface of the strip.
- The von Mises criterion of yield is adopted.
- The plastic deformation is a plane strain.
- Plane sections perpendicular to the direction of the rolling remain plane; stresses are uniformly distributed within each slab element.
- The contact arc is simplified as a string.

**Figure 1:**
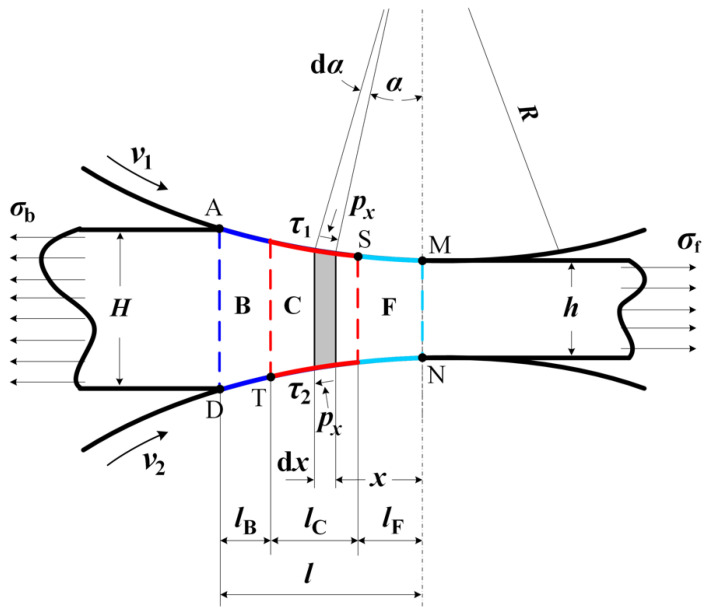
Schematic of deformation zone in asymmetrical strip rolling.

Figure 1 illustrates the schematic of a typical deformation zone in asymmetrical strip rolling. $v_{1}$ and $v_{2}$ are the peripheral speeds of the upper roll and lower roll, respectively, and $v_{1}>v_{2}$. The deformation zone is divided into three regions according to the direction of frictional stresses between the rolls and the strip. The region between the exit of the deformation zone and the neutral point is a forward-slip zone (F), the region between the upper neutral point and the lower neutral point is a cross-shear zone (C) and the region between the lower neutral point and the entrance of the deformation zone is a backward-slip zone (B).

The lengths of the forward-slip zone, cross-shear zone and backward-slip zone are denoted by $l_{F}$, $l_{C}$, $l_{B}$ and the length of contact $l=l_{F}$ + $l_{C}$ + $l_{B}$. The percentages of the three regions are expressed as $Q_{B}=l_{B}/l$, $Q_{C}=l_{C}/l$ and $Q_{F}=l_{F}/l$.

### 2.2. Rolling Pressure

**Figure 2:**
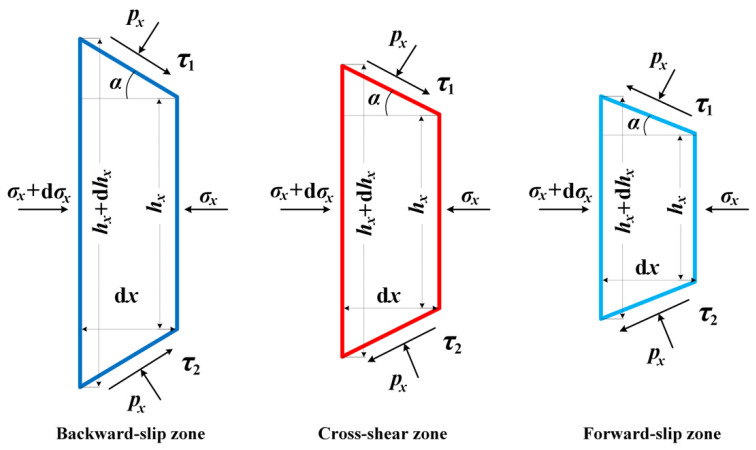
Stresses analysis on the slab in three deformation zones.

Stresses on a slab in three regions are illustrated in Figure 2. From the horizontal force equilibrium on a slab in the cross-shear zone, we obtain

(1) $$\left(\sigma_{x}+d\sigma_{x}\right)(h_{x}+dh_{x})-\sigma_{x}h_{x}-2p_{x}Rd\alpha \sin\alpha +\left(\tau_{1}-\tau_{2}\right)Rd\alpha \cos\alpha =0$$

where $\tau_{1}=p_{x}f_{1}$, $\tau_{2}=p_{x}f_{2}$ are friction stresses on the upper and lower surfaces of the strip, respectively.

In cold strip rolling, contact angle α is very small. From geometries shown in Figure 1 and Figure 2, it can be known that Rdα=dx/cosα, $dh_{x}=2dx\tan\alpha$.

The yield criterion for the plane strain can be expressed as

(2) $$p_{x}-\sigma_{x}=K$$

Referring to geometry in Figure 1, the thickness of a strip at a distance x from the center of the rolls can be expressed as

(3) $$h_{x}=h+\frac{\Delta h}{l}x$$

Substituting Equations (2) and (3) into Equation (1) and rearranging it, we have

(4) $$dp_{x}-\frac{dh_{x}}{h_{x}}\left(K-\delta_{2}p_{x}\;\right)=0$$

Integrating Equation (4) with respect to x, we obtain

(5) $$\frac{1}{\delta_{2}}\ln\left(\delta_{2}p_{x}-K\right)-\ln\frac{1}{h_{x}}=C_{C}^{*}$$

Using the same method, we obtain similar equations for slabs in the forward-slip zone and backward-slip zone,

(6) $$\frac{1}{\delta_{1}}\ln\left(\delta_{1}p_{x}+K\right)-\ln h_{x}=C_{F}^{*}$$

(7) $$\frac{1}{\delta_{1}}\ln\left(\delta_{1}p_{x}-K\right)+\ln h_{x}=C_{B}^{*}$$

where $\delta_{1}=\frac{\left(f_{1}+f_{2}\right)l}{\Delta h}$, $\delta_{2}=\frac{\left(f_{1}-f_{2}\right)l}{\Delta h}$. $C_{F}^{*}$, $C_{C}^{*}$ and $C_{B}^{*}$ are integral constants for the forward-slip zone, cross-shear zone and backward-slip zone, respectively.

The boundary conditions on the entrance and exit of the deformation zone are: $h_{x}=H$, $p_{x}=K-\sigma_{b}$ and $h_{x}=h$, $p_{x}=K-\sigma_{f}$. Substituting these boundary conditions into Equations (6) and (7), integral constants $C_{B}^{*}$ and $C_{F}^{*}$ are obtained. The rolling pressure of the forward-slip zone and backward-slip zone can be expressed as

(8) $$p_{F}=\frac{1}{\delta_{1}}\left[{\left(\frac{h_{x}}{h}\right)}^{\delta_{1}}\left(\delta_{1}\left(K-\sigma_{f}\right)+K\right)-K\right]$$

(9) $$p_{B}=\frac{1}{\delta_{1}}\left[{\left(\frac{H}{h_{x}}\right)}^{\delta_{1}}\left(\delta_{1}\left(K-\sigma_{b}\right)-K\right)+K\right]$$

The boundary conditions on the interface between the cross-shear zone and backward-slip are: $h_{x}=H-Q_{B}\Delta h$, $p_{x}=p_{B}$. Substituting these boundary conditions into Equation (5), integral constant $C_{C}^{*}$ can be obtained. Thus, rolling pressure of the cross shear zone is

(10) $$p_{C}=\frac{K}{\delta_{2}}{\left(\frac{H-Q_{B}\Delta h}{h_{x}}\right)}^{\delta_{2}}\left[\frac{\delta_{2}}{\delta_{1}}\left(\delta_{1}\varepsilon_{1}-1\right)\mu_{b}{}^{\delta_{1}}+\frac{\delta_{2}}{\delta_{1}}-1\right]+\frac{K}{\delta_{2}}$$

where $\mu_{b}=\frac{H}{H-Q_{B}\Delta h}$, $\mu_{f}=\frac{h+Q_{F}\Delta h}{h}$, $\varepsilon_{1}=\frac{K-\sigma_{b}}{K}$.

### 2.3. Three Region Percentages

On the interface between the cross-shear zone and forward-slip zone, $p_{C}=p_{F}$, the thickness of the strip on the interface is $h_{x}=h+Q_{F}\Delta h$. Combing Equation (8), Equation (10) and the boundary conditions, we obtain

(11) $$\mu_{f}{}^{\delta_{1}}\left(\delta_{1}\varepsilon_{2}+1\right)-1=i^{\delta_{2}}\left[\left(\delta_{1}\varepsilon_{1}-1\right){\left(\frac{\mu }{i}\right)}^{\delta_{1}}\frac{1}{\mu_{f}{}^{\delta_{1}}}+1-\frac{\delta_{1}}{\delta_{2}}\right]+\frac{\delta_{1}}{\delta_{2}}$$

By solving Equation (11), the percentages of the forward-slip zone, cross-shear zone and backward-slip zone can be obtained.

(12) $$Q_{F}=\frac{hX^{\frac{1}{\delta_{1}}}-h}{\Delta h}$$

(13) $$Q_{C}=\frac{\left(i-1\right)hX^{\frac{1}{\delta_{1}}}}{\Delta h}$$

(14) $$Q_{B}=\frac{H-ihX^{\frac{1}{\delta_{1}}}}{\Delta h}$$

(15) $$X=\frac{\left(i^{\delta_{2}}-\frac{\delta_{1}}{\delta_{2}}i^{\delta_{2}}+\frac{\delta_{1}}{\delta_{2}}+1\right)+\sqrt{{\left(i^{\delta_{2}}-\frac{\delta_{1}}{\delta_{2}}i^{\delta_{2}}+\frac{\delta_{1}}{\delta_{2}}+1\right)}^{2}+4\left(\delta_{1}\varepsilon_{2}+1\right)\left(\delta_{1}\varepsilon_{1}-1\right)i^{\delta_{2}}{\left(\frac{\mu }{i}\right)}^{\delta_{1}}}}{2\left(\delta_{1}\varepsilon_{2}+1\right)}$$

where $\varepsilon_{2}=\frac{K-\sigma_{f}}{K}$.

### 2.4. Roll Force

By integrating the normal rolling pressure along the arc of contact, the roll force can be obtained. Thus, the rolling force per unit width can be expressed as

(16) $$P=\int_{l-Q_{B}l}^{l}p_{B}dx+\int_{Q_{F}l}^{l-Q_{B}l}p_{C}dx+\int_{0}^{Q_{F}l}p_{F}dx$$

Substituting Equations (8)–(10) into Equation (16), we have

(17) $$\begin{array}{c} P=Kl\left[\frac{H\left(\delta_{1}\varepsilon_{1}-1\right)\left(\mu_{b}^{\delta_{1}-1}-1\right)}{\delta_{1}\Delta h\left(\delta_{1}-1\right)}+\frac{Q_{B}}{\delta_{1}}\right]+Kl\left\{\frac{H-Q_{B}\Delta h}{\delta_{2}\Delta h\left(\delta_{2}-1\right)}\left(\mu_{c}^{\delta_{2}-1}-1\right)\left[\frac{\delta_{2}}{\delta_{1}}\left(\delta_{1}\varepsilon_{1}-1\right)\mu_{b}^{\delta_{1}}+\frac{\delta_{2}}{\delta_{1}}-1\right]+\frac{Q_{C}}{\delta_{2}}\right\}\\ +Kl\left[\frac{h\left(\delta_{1}\varepsilon_{2}+1\right)\left(\mu_{f}^{\delta_{1}+1}-1\right)}{\delta_{1}\Delta h\left(\delta_{1}+1\right)}-\frac{Q_{F}}{\delta_{1}}\right] \end{array}$$

The roll force is $P_{T}=P*B_{0}$.

### 2.5. Roll Torque

The torque acting upon one roll can be obtained by integrating the torque acting upon the roll by the friction force of the unit area along the arc of contact. Frictional stress is $p_{F}f_{1}$, $p_{C}f_{1}$, $p_{B}f_{1}$ on the upper surfaces of the three regions illustrated in Figure 1, and $p_{F}f_{2}$, $p_{C}f_{2}$, $p_{B}f_{2}$ on the lower surfaces. The roll torque acting upon the upper work roll and lower work roll is

(18) $$T_{1}=R^{2}\int_{\alpha_{2}}^{\alpha_{l}}p_{B}f_{1}d\alpha +R^{2}\int_{\alpha_{1}}^{\alpha_{2}}p_{C}f_{1}d\alpha -R^{2}\int_{0}^{\alpha_{1}}p_{F}f_{1}d\alpha$$

(19) $$T_{2}=R^{2}\int_{\alpha_{2}}^{\alpha_{l}}p_{B}f_{2}d\alpha +R^{2}\int_{\alpha_{1}}^{\alpha_{2}}p_{C}f_{2}d\alpha -R^{2}\int_{0}^{\alpha_{1}}p_{F}f_{2}d\alpha$$

Substituting Equations (8)–(10) into Equations (18) and (19), and integrating Equations (18) and (19), we have

(20) $$\begin{array}{c} T_{1}=\frac{KRlf_{1}}{\delta_{1}\Delta h}\left[\frac{H\left(\delta_{1}\varepsilon_{1}-1\right)}{1-\delta_{1}}\left(1-{\mu_{b}}^{\delta_{1}-1}\right)+\Delta hQ_{B}\right]+\frac{KRlf_{1}\left(H-Q_{B}\Delta h\right)\left({\mu_{c}}^{\delta_{2}-1}-1\right)}{\delta_{2}\left(\delta_{2}-1\right)\Delta h}\left[\frac{\delta_{2}}{\delta_{1}}\left(\delta_{1}\varepsilon_{1}-1\right){\mu_{b}}^{\delta_{1}}+\frac{\delta_{2}}{\delta_{1}}-1\right]\\ +\frac{Q_{C}KRlf_{1}}{\delta_{2}}-\frac{KRlf_{1}}{\delta_{1}\Delta h}\left[\frac{h\left(\delta_{1}\varepsilon_{2}+1\right)}{\delta_{1}+1}\left({\mu_{f}}^{\delta_{1}+1}-1\right)-\Delta hQ_{F}\right] \end{array}$$

(21) $$\begin{array}{c} T_{2}=\frac{KRlf_{2}}{\delta_{1}\Delta h}\left[\frac{H\left(\delta_{1}\varepsilon_{1}-1\right)}{1-\delta_{1}}\left(1-{\mu_{b}}^{\delta_{1}-1}\right)+\Delta hQ_{B}\right]+\frac{KRlf_{2}\left(H-Q_{B}\Delta h\right)\left({\mu_{c}}^{\delta_{2}-1}-1\right)}{\delta_{2}\left(\delta_{2}-1\right)\Delta h}\left[\frac{\delta_{2}}{\delta_{1}}\left(\delta_{1}\varepsilon_{1}-1\right){\mu_{b}}^{\delta_{1}}+\frac{\delta_{2}}{\delta_{1}}-1\right]\\ +\frac{Q_{C}KRlf_{2}}{\delta_{2}}-\frac{KRlf_{2}}{\delta_{1}\Delta h}\left[\frac{h\left(\delta_{1}\varepsilon_{2}+1\right)}{\delta_{1}+1}\left({\mu_{f}}^{\delta_{1}+1}-1\right)-\Delta hQ_{F}\right] \end{array}$$

where $\mu_{c}=\frac{H-Q_{B}\Delta h}{h+Q_{F}\Delta h}$.

The total roll torque per unit width is $T=T_{1}+T_{2}$.

### 2.6. Roll Power

In rotational systems, power is the product of the torque and angular velocity. The roll power of one roll can be obtained by the product of torque acting upon the roll and roll angular velocity. Roll power of the upper roll and lower roll can be expressed as

(22) $$\begin{array}{cc} A_{1}=T_{1}\frac{v_{1}}{R}= & \frac{Klf_{1}v_{1}}{\delta_{1}\Delta h}\left[\frac{H\left(\delta_{1}\varepsilon_{1}-1\right)}{1-\delta_{1}}\left(1-{\mu_{b}}^{\delta_{1}-1}\right)+\Delta hQ_{B}\right]\\ & +\frac{Klf_{1}v_{1}\left(H-Q_{B}\Delta h\right)\left({\mu_{c}}^{\delta_{2}-1}-1\right)}{\delta_{2}\left(\delta_{2}-1\right)\Delta h}\left[\frac{\delta_{2}}{\delta_{1}}\left(\delta_{1}\varepsilon_{1}-1\right){\mu_{b}}^{\delta_{1}}+\frac{\delta_{2}}{\delta_{1}}-1\right]+\frac{Q_{C}Klf_{1}v_{1}}{\delta_{2}}\\ & -\frac{Klf_{1}v_{1}}{\delta_{1}\Delta h}\left[\frac{h\left(\delta_{1}\varepsilon_{2}+1\right)}{\delta_{1}+1}\left({\mu_{f}}^{\delta_{1}+1}-1\right)-\Delta hQ_{F}\right] \end{array}$$

(23) $$\begin{array}{cc} A_{2}=T_{2}\frac{v_{2}}{R}= & \frac{Klf_{2}v_{2}}{\delta_{1}\Delta h}\left[\frac{H\left(\delta_{1}\varepsilon_{1}-1\right)}{1-\delta_{1}}\left(1-{\mu_{b}}^{\delta_{1}-1}\right)+\Delta hQ_{B}\right]\\ & +\frac{Klf_{2}v_{2}\left(H-Q_{B}\Delta h\right)\left({\mu_{c}}^{\delta_{2}-1}-1\right)}{\delta_{2}\left(\delta_{2}-1\right)\Delta h}\left[\frac{\delta_{2}}{\delta_{1}}\left(\delta_{1}\varepsilon_{1}-1\right){\mu_{b}}^{\delta_{1}}+\frac{\delta_{2}}{\delta_{1}}-1\right]+\frac{Q_{C}Klf_{2}v_{2}}{\delta_{2}}\\ & -\frac{Klf_{2}v_{2}}{\delta_{1}\Delta h}\left[\frac{h\left(\delta_{1}\varepsilon_{2}+1\right)}{\delta_{1}+1}\left({\mu_{f}}^{\delta_{1}+1}-1\right)-\Delta hQ_{F}\right] \end{array}$$

In asymmetrical strip rolling, tensions exerted by the coiler motors also contributed to the plastic deformation of the strip. The tensile power per unit area can be obtained by the product of tensile stress and the velocity of the strip. The power of the coiler and uncoiler due to the tensions are

(24) $$A_{\mathrm{rf}}=\sigma_{f}hv_{h}$$

(25) $$A_{\mathrm{rb}}=-\sigma_{b}Hv_{H}$$

The total roll power per unit width required is $A_{T}=A_{1}+A_{2}+A_{\mathrm{rf}}+A_{\mathrm{rb}}$.

### 2.7. Friction Power

Friction power on the unit area is equivalent to the product of friction stress and relative slipping velocity, and total friction power can be obtained by integrating it on the whole friction surface. Friction power on the upper surface of the strip in the deformation zone is

(26) $$A_{f1}=\int_{0}^{l_{F}}p_{F}f_{1}\left(\frac{v_{x}}{\cos\alpha }-v_{1}\right)dx+\int_{l_{F}}^{l_{F}+l_{C}}p_{C}f_{1}\left(v_{1}-\frac{v_{x}}{\cos\alpha }\right)dx+\int_{l_{F}+l_{C}}^{l}p_{B}f_{1}\left(v_{1}-\frac{v_{x}}{\cos\alpha }\right)dx$$

In cold rolling of a thin strip, the contact angle α is very small; thus, cosα≈1. The mass flow relationship in the deformation zone can be expressed as $h_{x}v_{x}=h_{1}v_{1}=h_{2}v_{2}$. Substituting Equations (8)–(10) into Equation (26), we obtain

(27) $$\begin{array}{cc} A_{f1}=\frac{Klf_{1}}{\delta_{1}\Delta h} & \left[\frac{h_{2}v_{2}\left(\delta_{1}\varepsilon_{2}+1\right)\left(\mu_{f}^{\delta_{1}}-1\right)}{\delta_{1}}-h_{2}v_{2}\ln\mu_{f}-\frac{hv_{1}\left(\delta_{1}\varepsilon_{2}+1\right)\left(\mu_{f}^{\delta_{1}+1}-1\right)}{\delta_{1}+1}+v_{1}\left(h_{1}-h\right)\right]\\ & +\frac{Klf_{1}}{\delta_{2}\Delta h}\left\{\frac{h_{2}v_{1}\left(\mu_{c}^{\delta_{2}-1}-1\right)}{\delta_{2}-1}\left[\frac{\delta_{2}}{\delta_{1}}\left(\delta_{1}\varepsilon_{1}-1\right)\mu_{b}^{\delta_{1}}+\frac{\delta_{2}}{\delta_{1}}-1\right]+v_{1}\left(h_{2}-h_{1}\right)\right.\\ & \left.-\frac{h_{2}v_{2}\left(\mu_{c}^{\delta_{2}}-1\right)}{\delta_{2}}\left[\frac{\delta_{2}}{\delta_{1}}\left(\delta_{1}\varepsilon_{1}-1\right)\mu_{b}^{\delta_{1}}+\frac{\delta_{2}}{\delta_{1}}-1\right]-h_{2}v_{2}\ln\mu_{c}\right\}\\ & +\frac{Klf_{1}}{\delta_{1}\Delta h}\left[\frac{Hv_{1}\left(\delta_{1}\varepsilon_{1}-1\right)\left(\mu_{b}^{\delta_{1}-1}-1\right)}{\delta_{1}-1}+v_{1}\left(H-h_{2}\right)-\frac{h_{2}v_{2}\left(\delta_{1}\varepsilon_{1}-1\right)\left(\mu_{b}^{\delta_{1}}-1\right)}{\delta_{1}}\right.\\ & -h_{2}v_{2}\ln\mu_{b}] \end{array}$$

Friction power on the lower surface of the strip in the deformation zone can be obtained using the same method.

(28) $$\begin{array}{cc} A_{f2}=\frac{Klf_{2}}{\delta_{1}\Delta h} & \left[\frac{h_{2}v_{2}\left(\delta_{1}\varepsilon_{2}+1\right)\left(\mu_{f}^{\delta_{1}}-1\right)}{\delta_{1}}-h_{2}v_{2}\ln\mu_{f}-\frac{hv_{2}\left(\delta_{1}\varepsilon_{2}+1\right)\left(\mu_{f}^{\delta_{1}+1}-1\right)}{\delta_{1}+1}+v_{2}\left(h_{1}-h\right)\right]\\ & +\frac{Klf_{2}}{\delta_{2}\Delta h}\left\{-\frac{h_{2}v_{1}\left(\mu_{c}^{\delta_{2}-1}-1\right)}{\delta_{2}-1}\left[\frac{\delta_{2}}{\delta_{1}}\left(\delta_{1}\varepsilon_{1}-1\right)\mu_{b}^{\delta_{1}}+\frac{\delta_{2}}{\delta_{1}}-1\right]+v_{2}\left(h_{2}-h_{1}\right)\right.\\ & \left.+\frac{h_{2}v_{2}\left(\mu_{c}^{\delta_{2}}-1\right)}{\delta_{2}}\left[\frac{\delta_{2}}{\delta_{1}}\left(\delta_{1}\varepsilon_{1}-1\right)\mu_{b}^{\delta_{1}}+\frac{\delta_{2}}{\delta_{1}}-1\right]-h_{2}v_{2}\ln\mu_{c}\right\}\\ & +\frac{Klf_{2}}{\delta_{1}\Delta h}\left[\frac{Hv_{2}\left(\delta_{1}\varepsilon_{1}-1\right)\left(\mu_{b}^{\delta_{1}-1}-1\right)}{\delta_{1}-1}+v_{2}\left(H-h_{2}\right)-\frac{h_{2}v_{2}\left(\delta_{1}\varepsilon_{1}-1\right)\left(\mu_{b}^{\delta_{1}}-1\right)}{\delta_{1}}\right.\\ & -h_{2}v_{2}\ln\mu_{b}] \end{array}$$

The friction power per unit width can be expressed as $A_{f}=A_{f1}+A_{f2}$.

### 2.8. Energy Consumption in Asymmetrical Rolling

The energy consumption in asymmetrical strip rolling refers to the work consumed during the process of rolling a strip of a certain size to target thickness. In cold strip manufacturing, the main energy-consuming process is rolling. Therefore, only energy consumption in the rolling process of asymmetrical strip rolling is investigated in this work. The energy consumption in the rolling process mainly consists of energy consumption in rolling, energy consumption of auxiliary equipment and energy consumption due to friction in the drive system. To simplify the analysis, only the energy consumption in rolling is taken into account in this work.

The energy consumption in rolling is equivalent to the work completed by the motors of the rolling mill and the motors of the coiler and uncoiler. The energy consumption in one pass can be expressed as

(29) $$W_{T}=W_{1}+W_{2}+W_{\mathrm{rf}}+W_{\mathrm{rb}}$$

where $W_{1}=\frac{A_{1}B_{0}t}{60000}$, $W_{2}=\frac{A_{2}B_{0}t}{60000}$, $W_{\mathrm{rf}}=\frac{A_{\mathrm{rf}}B_{0}t}{60000}$, $W_{\mathrm{rb}}=\frac{A_{\mathrm{rb}}B_{0}t}{60000}$.

The work performed in the deformation during plastic working is equal to the work of external forces. In a rolling pass, rolling work ($W_{T}$) is equivalent to the sum of the plastic deformation work of the strip ($W_{d}$) and the friction work on the contact surface between the strip and rolls ($W_{f}$).

(30) $$W_{T}=W_{d}+W_{f}$$

Friction work $W_{f}$ consists of $W_{f1}$ (on the upper surface) and $W_{f2}$ (on the lower surface)

(31) $$W_{f}=W_{f1}+W_{f2}$$

where $W_{f1}=\frac{A_{f1}B_{0}t}{60000}$, $W_{f2}=\frac{A_{f2}B_{0}t}{60000}$.

The proportion of friction work ($Q_{f}$) and proportion of plastic deformation work ($Q_{d}$) can be expressed as $Q_{f}=\frac{Q_{f}}{W_{T}}\times 100\%$ and $Q_{d}=\frac{Q_{d}}{W_{T}}\times 100\%$, respectively.

## 3. Results and Discussion

Several asymmetrical strip rolling experiments have been conducted on the asymmetrical mill, with a work roll diameter of 90 mm, to verify the validity of the proposed models. The lower roll served as the slow roll and was set to a peripheral velocity of 4.0 m/min, while the peripheral velocity of the upper roll varied with the speed ratio. Emulsion was used as a lubricant. The 430 stainless steel strips, with 80.0 mm in width, were rolled from 0.150 mm to 0.105 mm under different speed ratios. By fitting the tensile test data of 430 stainless steel strips with different reduction ratios, the plane deformation resistance can be expressed as

(32) $$K=407.9+588.6{\bar{\varepsilon }}^{0.718}$$

where $\bar{\varepsilon }=0.4\left(1-\frac{H}{H_{0}}\right)+0.6\left(1-\frac{h}{H_{0}}\right)$.

**Figure 3:**
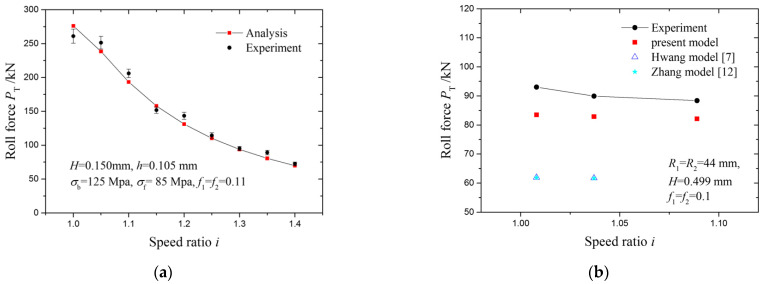
Comparisons of model-calculated roll force with: (**a**) experimental results; (**b**) experimental results and results of literature models.

The model-calculated roll force is compared with the experiment-measured roll force. A comparison of roll force between experimental measurement values and those calculated by the models is illustrated in Figure 3a. It was found that both the experimental and the calculated roll force decreased with an increasing speed ratio. The maximum error and RMS error between the experimentally measured roll forces and calculated ones are 9.7% and 5.9%, respectively. The model-calculated forces are also compared with the results of models from the literature in Figure 3b. Models from the literature cannot finish calculations in the third point. All model-calculated roll forces are less than experimental values, but the present model has better accuracy than models from the literature. Data used in the calculation are listed in Table 1.

The proposed models are used to calculate rolling pressure, roll force, roll torque and roll power, as well as friction work and plastic deformation work in asymmetrical strip rolling. A work roll diameter of 88 mm, and 430 stainless steel strips of initial length *L*_0_ = 1000 m and initial width *B*_0_ = 100 mm, are used in the analysis. From the calculation results, the effects of rolling parameters on friction work and plastic deformation work are analyzed.

**Figure 4:**
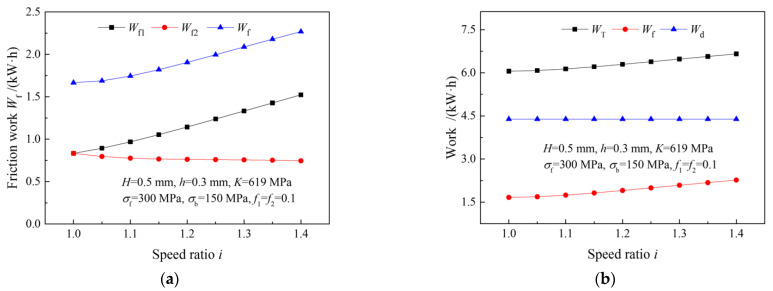
Effects of speed ratio on: (**a**) friction work, (**b**) plastic deformation work and rolling work, in a single pass.

Figure 4 illustrates the effects of the speed ratio on friction work, plastic deformation work and rolling work in a single rolling pass with the same reduction ratio. It can be seen that the upper friction work increases significantly with an increasing speed ratio, but the lower friction work only increases slightly. This is because the percentage of cross-shear zone increases with an increasing speed ratio. As a result, the upper and lower neutral points move toward the exit and the entrance of the deformation zone, respectively, thus increasing the slipping distance between the upper roll and the strip. Therefore, the friction work increases. The plastic deformation work remains unchanged under a different speed ratio because the deformation degree is the same.

**Figure 5:**
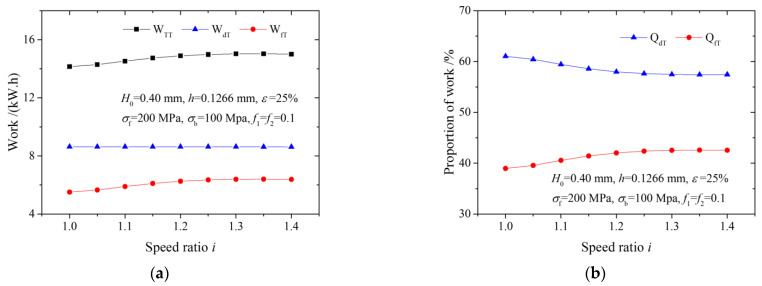

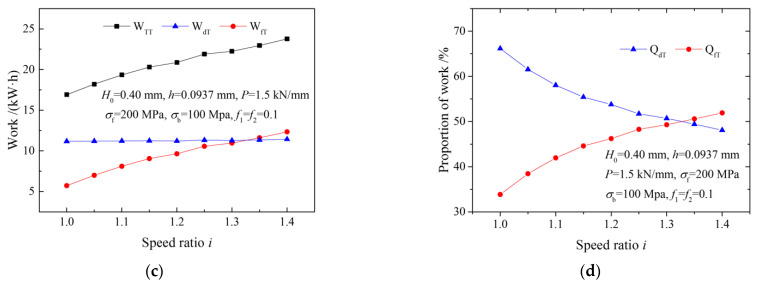
Sum of works and proportions of friction work and plastic deformation work in multi-pass rolling: Work (**a**), proportion of friction work and plastic deformation work (**b**) in a five-pass rolling with same reduction ratio; work (**c**), proportion of friction work and plastic deformation work (**d**) in multi-pass rolling with same roll force.

Figure 5 illustrates the effects of the speed ratio on total friction work, total plastic deformation work, total rolling work, the proportion of total friction work and the proportion of total plastic deformation work in multi-pass rolling. The curves in Figure 5a,b were obtained from five-pass rolling with the same reduction ratio, while the curves in Figure 5c,d were obtained from multi-pass rolling with the same roll force. It is seen that both the total friction work and the total rolling work increase as the speed ratio increases, but the total plastic deformation work remains constant, as in Figure 5a,c. Owing to the same level of accumulated reduction, the total plastic deformation work is not affected by the speed ratio. The sum of accumulated relative velocity between rolls and strips and the sum of the slipping distance increase with an increasing speed ratio. Consequently, the total friction work increases, leading to an increase in the total rolling work. Correspondingly, the proportion of total friction work gradually increases, and the proportion of total plastic deformation work gradually decreases with an increasing speed ratio, as shown in Figure 5b,d. The proportion of total friction work exceeds the proportion of total plastic deformation work at a certain speed ratio.

**Figure 6:**
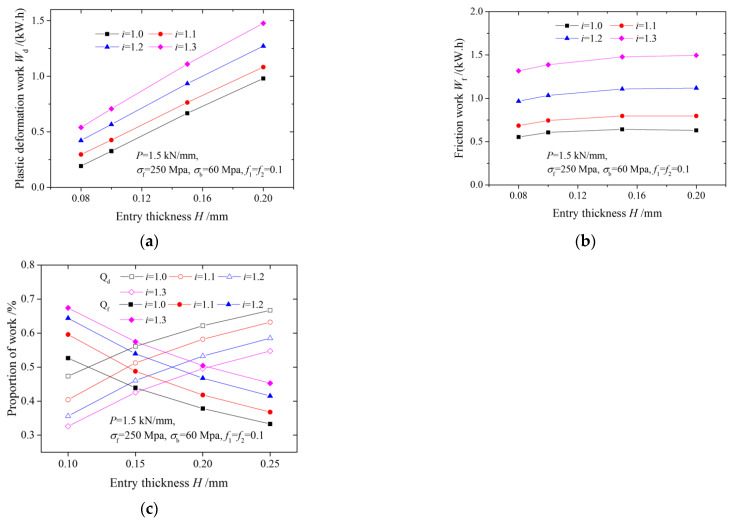
Variation of (**a**) plastic deformation work, (**b**) friction work, and (**c**) proportions of plastic deformation work and friction work with entry thickness.

Figure 6 illustrates the effects of the speed ratio and entry thickness on friction work, plastic deformation work and its proportions. According to Figure 6, we know that the plastic deformation work increases with both an increasing speed ratio and an increasing entry thickness. This is because both an increase in entry thickness and an increase in speed ratio increase the magnitude of deformation, thus resulting in an increase in plastic deformation work. Friction work increases with an increasing speed ratio for the same entry thickness, and also increases with increasing thickness for the same speed ratio. As the speed ratio increases, the percentage of cross-shear zone increases, and accumulated relative velocity between rolls and strips consequently increases. As a result, friction work increases.

**Figure 7:**
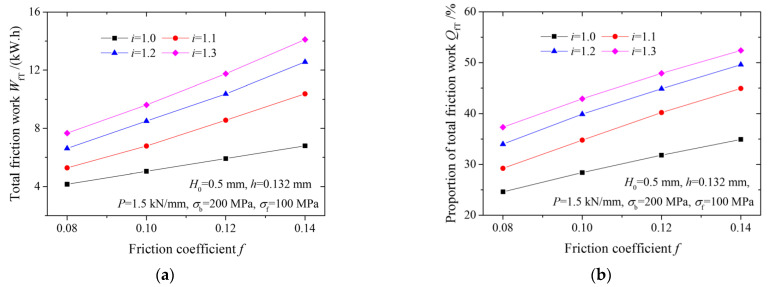
Variation of (**a**) total friction work and (**b**) proportion of total friction work with friction coefficient.

Figure 7 illustrates the effects of the friction coefficient and speed ratio on total friction work and its proportions in total rolling work. As the speed ratio increases, both the total friction work and the proportion of total friction work increase. The total friction work and the proportion of total friction work also increase with an increased friction coefficient.

**Figure 8:**
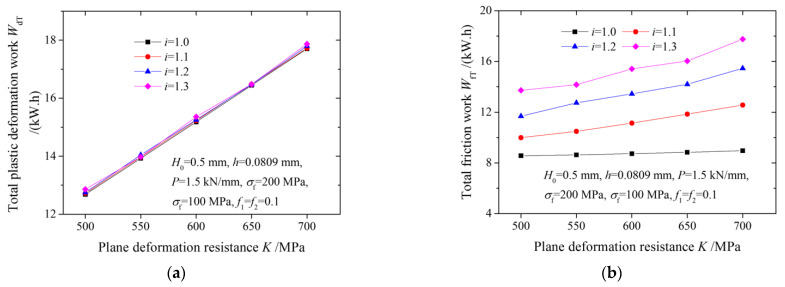

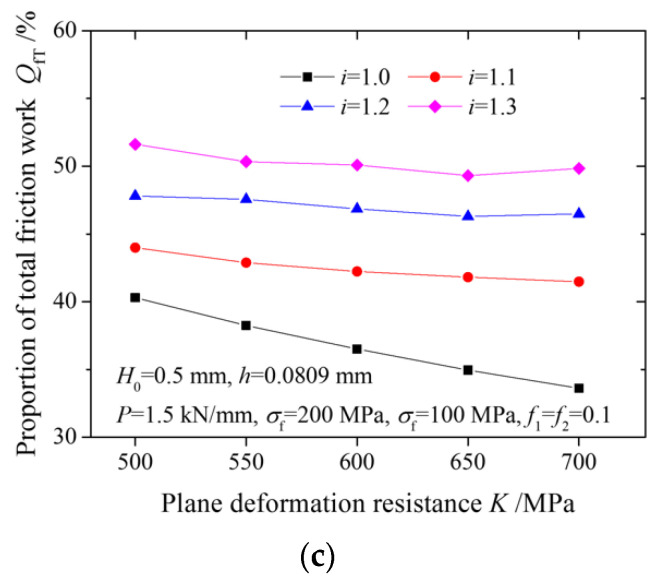
Variation of (**a**) total plastic deformation work, (**b**) total friction work, and (**c**) proportion of total friction work with plane deformation resistance.

The curves in Figure 8 show the effects of plane deformation resistance and the speed ratio on the total plastic deformation work, total friction work and proportion of total friction work in multi-pass rolling. Given that the deformation force increases with an increase in plane deformation resistance, and the levels of accumulated reduction are the same, the total plastic deformation work increases with an increase in plane deformation resistance. The increase in plane deformation resistance causes the percentage of the cross-shear zone to increase, thus increasing the total friction work. The proportion of total friction work decreases with an increase in plane deformation resistance because the increment rate in the total plastic deformation work is greater than in the total friction work.

**Figure 9:**
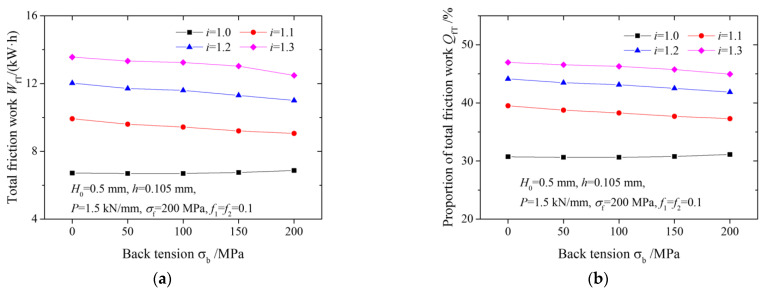
Variation of (**a**) total friction work and (**b**) proportion of total friction work with back tension.

Figure 9 shows the effects of the speed ratio on the total friction work and the proportions of total friction work under various back tensions. The percentage of the cross-shear zone decreases with the increase in back tension, resulting in a decrease in the relative slip between the strip and roll. Therefore, the total friction work decreases slightly with an increasing back tension.

The effects of front tension are similar to that of back tension.

**Figure 10:**
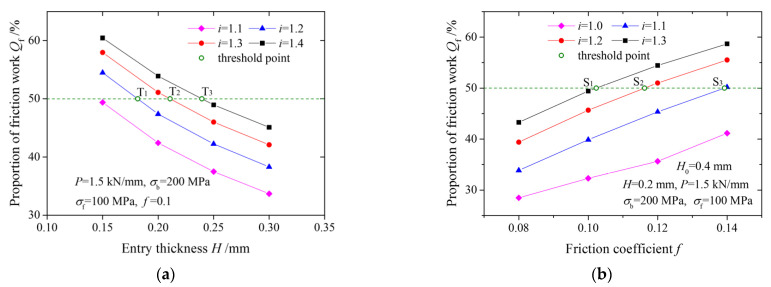
Variation of threshold point and proportions of friction work with: (**a**) entry thickness; (**b**) friction coefficient.

The effects of the entry thickness and the friction coefficient on the proportions of friction work are shown in Figure 10. There is a special line on which the proportion of friction work is equal to 50%; in other words, the proportion of friction work is the same as the proportion of plastic deformation work. At points T1, T2, and T3 in Figure 10a, which are called the threshold points, the curve is divided into two parts, and the friction work is greater than the plastic deformation work on the left part. On the contrary, at points S1, S2, and S3 in Figure 10b, the friction work is greater than the plastic deformation work on the right part.

The threshold point is related to friction conditions in the deformation zone and the deformation degree; it is affected by parameters such as thickness, the friction coefficient, the speed ratio, front and back tension, deformation resistance and the reduction ratio.

## 4. Conclusions

Mathematic models based on the slab method are built to calculate the rolling pressure, roll force, roll torque, friction power, rolling power and rolling work for the three typical region deformation zone configurations in the asymmetrical rolling of a thin strip. The effects of rolling parameters on energy consumption (friction work and plastic deformation work) are investigated by analyzing computed results. The following conclusions are obtained.

- In all cases, an increase in speed ratio leads to the increase in friction work, thus resulting in the increase in the rolling work, increase in the proportion of friction work and decrease in the proportion of plastic deformation work.
- The increase in entry thickness, deformation resistance of the strip and the friction coefficient cause friction work and rolling work to increase. The increase in entry thickness and deformation resistance also leads to an increase in plastic deformation work.
- The increase in the friction coefficient leads to an increase in the proportion of friction work. The increase in deformation resistance of the strip, front tension and back tension result in a decrease in the proportion of friction work.
- In conditions of a very thin strip being rolled with a large speed ratio, the proportion of friction work exceeds that of plastic deformation work. The concept of the threshold point, at which the friction work is equal to the plastic deformation work, was proposed for this case.
- The comparison between the roll forces obtained from the experimental measurement and calculation results shows a maximum error of 9.7% and an RMS error of 5.9%.

## Figures and Tables

**Table 1:** Data for roll force calculation.

Entry Thickness, mm	Reduction, %	Back Tension, MPa	Front Tension, MPa	Speed Ratio	Yield Shear Strength, MPa	Experimental Force, kN
0.499	14.33	111.35	85.92	1.008	257.0	93.07
	14.79	111.78	99.72	1.037	258.2	89.95
	15.87	116.73	98.52	1.089	261.2	88.40

## Data Availability

Not applicable.
